# Medical College of South Carolina, at Charleston

**Published:** 1858-03

**Authors:** 


### Medical College of South Carolina, at Charleston.

We have received a catalogue of the Students at the above Institution, for the Session of 1857–'58, from which we learn that they had in attendance from South Carolina 139; North Carolina, 19; Alabama, 24; Mississippi, 12; Florida, 10; Georgia, 7; Louisiana, 1; Texas, 1; California, 1; New York, 1; Cuba, 1—making in the aggregate 216, connected with which we notice the remarkable fact, that with one exception, they were all undergraduates. This Institution is controlled by *gentlemen* for whom we have great respect, being far too elevated to entertain the petty and discreditable jealousies which are exhibited in some other quarters of the *South.* Long may it continue to flourish, say we.

